# Structure Characterization and Potential Probiotic Effects of Sorghum and Oat Resistant Dextrins

**DOI:** 10.3390/foods11131877

**Published:** 2022-06-24

**Authors:** Wenwen Chen, Ting Zhang, Qi Ma, Yingying Zhu, Ruiling Shen

**Affiliations:** 1 College of Food and Bioengineering, Zhengzhou University of Light Industry, No. 166 Kexue Road, Zhengzhou 450002, China; chenwenwen1112@163.com (W.C.); zhangtingppll@163.com (T.Z.); maqi6274@163.com (Q.M.); 2018072@zzuli.edu.cn (Y.Z.); 2 Henan Key Laboratory of Cold Chain Food Quality and Safety Control, No. 166 Kexue Road, Zhengzhou 450002, China; 3 Collaborative Innovation Center of Food Production and Safety, No. 166 Kexue Road, Zhengzhou 450002, China

**Keywords:** sorghum, oat, resistant dextrin, physical properties, prebiotic

## Abstract

Resistant dextrins (RDs) were prepared from sorghum and oat starches to determine their molecular structure, physicochemical properties, digestibility and prebiotics effect in vitro. The results showed that the particle size of sorghum resistant dextrin (SRD) and oat resistant dextrin (ORD) was significantly smaller than their respective starches. They formed a block structure, and lost the original A-type structure. In addition, SRD and ORD had good thermal stability, solubility (>90%) and enzymatic hydrolysis resistance (digestibility < 5%). The potential probiotic effects of ORD and SRD were studied by measurement of their promoting effects on the growth of *Lactiplantibacillus plantarum*, *Lactobacillus acidophilus* and *Lactobacillus delbrueckii*. For *Lactiplantibacillus plantarum* and *Lactobacillus acidophilus*, the promoting effect of ORD was the best (*p* < 0.05), and the counts increased by 8.89 and 8.74 log CFU/mL, respectively, compared with the control. For *Lactobacillus delbrueckii*, SRD was most effective, increasing the counts by 8.72 log CFU/mL compared with the control. These characteristics make SRD and ORD suitable for use as soluble dietary fiber and prebiotics in beverages and the excipients of low-glycemic-index products.

## 1. Introduction

Resistant dextrins (RDs) may be produced by hydrolysis of starch with enzymes or acids in water, as well as by dextrinization or pyroconversion, or heating starch in its dry form [1]. These reactions produce α-1,2, α-1,3, β-1,2 and β-1,6 glycosidic linkages responsible for an increase in α-amylase resistance [2,3]. There are numerous applications of RDs is, due to their physiological functions (i.e., regulate blood sugar, reduce blood lipids and improve the composition of intestinal flora) and excellent processing properties (i.e., good solubility, low viscosity and good thermal stability) [4,5,6].

A variety of starch sources are used to produce RDs such as those from corn, potato, wheat, oat, sorghum, cassava, and tapioca. The molecular weight profiles and contents of indigestible fractions of RDs depend on botanical origin [7]. RDs with lower molecular weights, more highly branched structures, and increased levels of indigestible linkages possess higher hypoglycemic activities [8]. Laurentin et al. [7] showed that major carbohydrate fractions (52–63%) of RDs obtained from cassava, cocoyam, lentil, maize and sagu root starches had molecular weights ranging from 8 to 105 kDa, while the molecular weight of the major fraction found in the sorghum-derived RD was greater than 105 kDa. Sorghum starch is less susceptible to pyrodextrinization than cassava, cocoyam, lentil, sagu root starches. Weil et al. [9] found that RDs from waxy tapioca starch produced at 170 °C/4 h had a 5% higher total indigestible carbohydrate than RDs from normal tapioca starch (45.2% and 40.4%, respectively). The low-molecular-weight indigestible carbohydrate content at this condition was also higher for waxy tapioca starch than normal tapioca starch (40.6% and 34.9%, respectively) [10]. A previous study found that the small size of starch granules, high lipid content, the small chain length of amylose and high relative crystallinity are special characteristics of oat starch [11]. The amylose content and degree of branching of amylopectin in oat starch were significantly higher than the corresponding parameters in rice starch. Sorghum is an underutilized crop which can replace corn starch at the industrial level after enhancement of its functional properties [12]. However, the correlation between the structure and properties of oat resistant dextrin (ORD) and sorghum resistant dextrin (SRD) and their starch needs to be further explored and research on prebiotics of ORD and SRD is limited. Therefore, our research aimed (1) to prepare RD from sorghum and oat starch, (2) to characterize their structure and physicochemical properties, and (3) to study their in vitro digestion characteristics and potential prebiotic effects. Data from this research provide a better understanding of the correlation between the structure and properties of starch sources and resistant dextrins, as well as the potential prebiotic effects of ORD and SRD. This study also promotes the research and development of sorghum and oat functional products (especially low-glycemic-index products and prebiotic-rich beverages), and promotes the comprehensive utilization of oat and sorghum.

## 2. Materials and Methods

### 2.1. Materials

Sorghums and oats were obtained from the Shanxi breeding Institute (Shanxi, China). The OS and SS were prepared in the laboratory with a purity of 91.51 ± 1.45%, and 92.13 ± 1.23% (AACC 76.13), respectively. Thermostable α-amylase from Bacillus subtilis was purchased from Shanghai Yuan ye Bio-Technology Co., Ltd. (Shanghai, China), Pepsin and glucosidase were purchased from Sigma Chemical Co. (St. Louis, MO, USA). *Lactiplantibacillus plantarum* (*Lactobacillus plantarum* subsp. Plantarum, GDMCC 1.1797), *Lactobacillus acidophilus* (GDMCC 1.321) and *Lactobacillus delbrueckii* (*Lactobacillus delbrueckii* subsp. Bulgaricus, GDMCC 1.155) were purchased from Guangdong Microbial Culture Collection Center (Guangdong, China). All the other chemicals were of analytical grade.

### 2.2. Preparation of Samples

RDs were prepared by the method of Barczynska et al. [13] with some modifications. Briefly, the starch was sprayed with HCl solution (1%, *v*/*v*). After 1 h, the starch was dried at pyrolysis temperature until the moisture content was below 5%, and pyrodextrin was obtained. Then, the pyrodextrin was dispersed in water to obtain a crude solution (25%, *w*/*v*). The crude solution was adjusted to pH 6.0 by adding a NaOH solution (0.4%, *w*/*v*) and reacted with thermostable α-amylase (0.1%, *v*/*v*) at 95 °C for 2 h. Then, the pH was adjusted to 10.0. The alkaline protease (0.05%, *m*/*v*) was added to the crude solution and reacted at 60 °C for 1 h. After that, the pH was adjusted to pH 4.5 and glucosidase was added to the mixture kept at 60 °C for 36 h. After decolorization and filtration, the final products were concentrated by a rotary evaporator. The samples were freeze-dried after being precipitated by ethanol. The amount of acid, pyrolysis temperature and pyrolysis time in the preparation of ORD and DRD were as follows: ORD: 12%, 190 °C, and 90 min; SRD: 10%, 180 °C, and 70 min. The purity of the RDs was 89.69 ± 1.15% and 88.23 ± 1.24% (AOAC 985.29), respectively.

### 2.3. Structure Characterization

#### 2.3.1. Scanning Electron Microscopy (SEM)

The morphological characteristics of the RDs were visualized by SEM (JSM-6490LV FEI Instruments, Hillsboro, OR, USA) under a 20 KV acceleration voltage. The sample was fixed on the sample holder, sputter coated with gold, and the magnification was adjusted to 2000× and 5000×, respectively, to observe the morphology [14].

#### 2.3.2. Fourier Transform Infrared Spectroscopy (FTIR)

An infrared spectrogram was conducted using FTIR (Vertex70, Bruker Ltd., Billica, MA, USA). The tablets were prepared by 1.0 mg samples (dry basic) mixed with potassium bromide at a ratio of 1:100, then they were scanned at 4000 to 400 cm^−1^ using the infrared spectrometer, and deconvolution was performed at the range of 1200 to 800 cm^−1^ [15].

#### 2.3.3. X-ray Diffraction (XRD)

XRD patterns were recorded by da Silva et al. [16] with some modifications. The scanning region of the diffraction angle (2θ) was 5° to 60° with a voltage of 40 kV using an XRD (PW-1710, Philips, Amsterdam, The Netherlands).

### 2.4. Physicochemical Properties

#### 2.4.1. Hydration Properties

The water solubility index (WSI) and the swelling power (SP) were analyzed at 4, 25, 37 and 100 °C [17]. The preparation of suspensions was separately carried out with 1 g of the RD per 80 mL of the solution. Suspensions were, respectively, stirred for 30 min at different temperatures and centrifugation (2683× *g*, 20 min). The supernatants were then oven-dried (105 °C) until the weight was constant. All analyses were performed in triplicate. The water solubility index (WSI, Equation (1)) and the swelling power (SP, Equation (2)) were calculated as follows:

(1) $$\mathrm{WSI}\left(\%\right)=\frac{\mathrm{Dry}\;\mathrm{supernatant}\;\mathrm{weight}}{\mathrm{Dry}\;\mathrm{sample}\;\mathrm{weight}}\times 100$$

(2) $$\mathrm{SP}=\frac{\mathrm{Wet}\;\mathrm{sediment}\;\mathrm{weight}}{\mathrm{Dry}\;\mathrm{sample}\;\mathrm{weight}\times \left[1-\mathrm{WSI}\left(\%\right)/100\right]}$$

#### 2.4.2. Differential Scanning Calorimetry (DSC)

The thermal properties of the samples were analyzed by DSC (Q100, TA Instruments, New Castle, DE, USA). The 3 mg samples were mixed with 7 mg distilled water in a DSC pan and equilibrated overnight at room temperature. The pan was heated at a rate of 10 °C/min from 20 to 120 °C and an empty DSC pan was used as a reference.

### 2.5. In Vitro Digestibility

The extent of in vitro starch hydrolysis was treated as described by Yu et al. [18] with some modifications. The suspension was made by adding 6 mL deionized water to 100 mg samples (dry base). After gelatinizing for 10 min in a boiling water bath, the suspension was immediately placed in a 37 °C water bath to reach equilibrium temperature. Then, the suspension was incubated for 2 h with simulated gastric juice (pH = 2) and concussion at 37 °C. The suspensions were then neutralized with 0.02 M NaOH, mixed with 3 mL amylase-amyloglucosidase solution, and shaken for 3 h at 37 °C. Aliquots (0.5 mL) were taken and 4.5 mL of 0.3 M Na_2_CO_3_ solution were added to inactivate the enzymes, and then centrifuged (1006*× g*, 5 min). The content of free reducing sugar in the supernatant was determined by the 3,5-dinitrosali-cyclic acid (DNS) assay. The hydrolysis rate is calculated as follows:

(3) $$\mathrm{Hydrolysis}\;\mathrm{rate}\;\left(\%\right)=\frac{\mathrm{reducing}\;\mathrm{sugar}\;\mathrm{content}}{\mathrm{total}\;\mathrm{starch}}\times 100\%$$

### 2.6. Probiotics Effect In Vitro

The prebiotic effects of SRD and ORD were evaluated by performing in vitro fermentation, using *Lactiplantibacillus plantarum*, *Lactobacillus acidophilus* and *Lactobacillus delbrueckii* [19]. Two kinds of RD solutions obtained after simulated in vitro digestion were used to replace the carbon source in MRS solid medium and adjusted to a final concentration of 0, 4, 8, 12, 16 and 20 mg/mL. MRS medium with 0 mg/mL RD solution was used as the control. 100 μL diluted suspension was added to MRS liquid medium. Then, the medium was placed at 37 °C for anaerobic culture for 48 h and the total number of colonies was recorded by plate counting method.

The probiotics with the best culture effect were then selected. A volume of 1 mL of bacterial suspension was inoculated into 100 mL of different mediums (add the same amount (1%) to the liquid medium with the best proportion of the RD selected from the two groups) in triplicate, and then incubated at 37 °C for 48 h. Samples were collected at intervals and the growth rates were examined by measuring the pH and optical density at 600 nm (OD_600_).

### 2.7. Statistical Analysis

All experiments were conducted in triplicate and the results were expressed as the mean ± standard deviation. Origin 7.5 (Origin Lab, Northampton, MA, USA) was used to organize and draw the data. Statistical analyses were performed by the statistical software SPSS version 22.0 (IBM Corp., Armonk, NY, USA) using ANOVA and Tukey’s post hoc test (*p* < 0.05).

## 3. Results and Discussion

### 3.1. Granule Morphology

**Figure 1:**
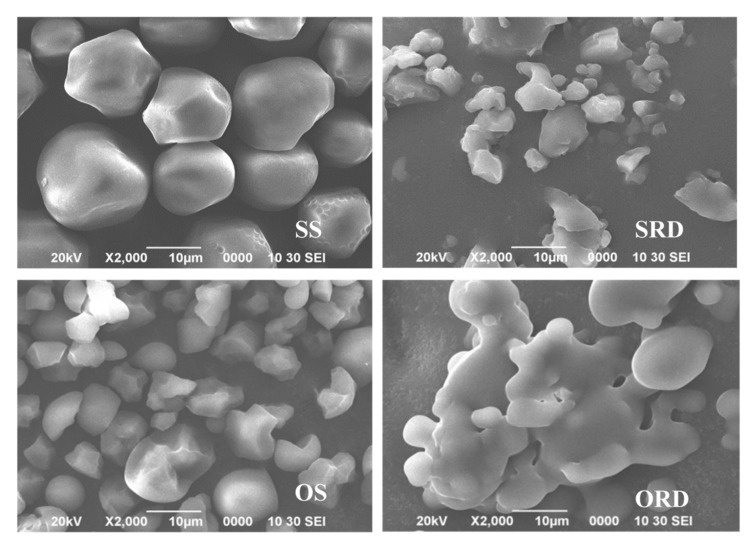
Scanning electron microscopy (2000×) of sorghum/oat starch and sorghum/oat resistant dextrin. SS: sorghum starch; SRD: sorghum resistant dextrin; OS: oat starch; ORD: oat resistant dextrin.

The morphological characteristics of the samples are shown in Figure 1 at a magnification of 2000×. The granular structure of SS was more regular than that of OS—mostly round, oval or polygonal—and the surface was smooth. The granule of OS was polyhedral with a regular surface, no crack and stomata, which was consistent with the results obtained by Zhang et al., who reported the irregular geometric, oval, angular and uneven particles of OS [20]. The particle size of the two prepared RDs was significantly smaller than their respective starch. SRD and ORD were mostly block structures or irregular structures formed by small molecules gathered together. This may be because the original molecular structure of starch was destroyed by high-temperature acidolysis and so, to a certain extent, the repolymerization of small molecules and the intermolecular glycosyl transfer reaction were promoted simultaneously, thus forming a more complex and irregular structure, accompanied by the production of small molecular sugars. Similar findings were noted in the preparation of RDs from lentil and corn starch [7]. Adhesion between particles of ORD was observed, which may be due to the destruction of its surface structure under harsh preparation conditions.

### 3.2. FTIR Spectra

**Figure 2:**
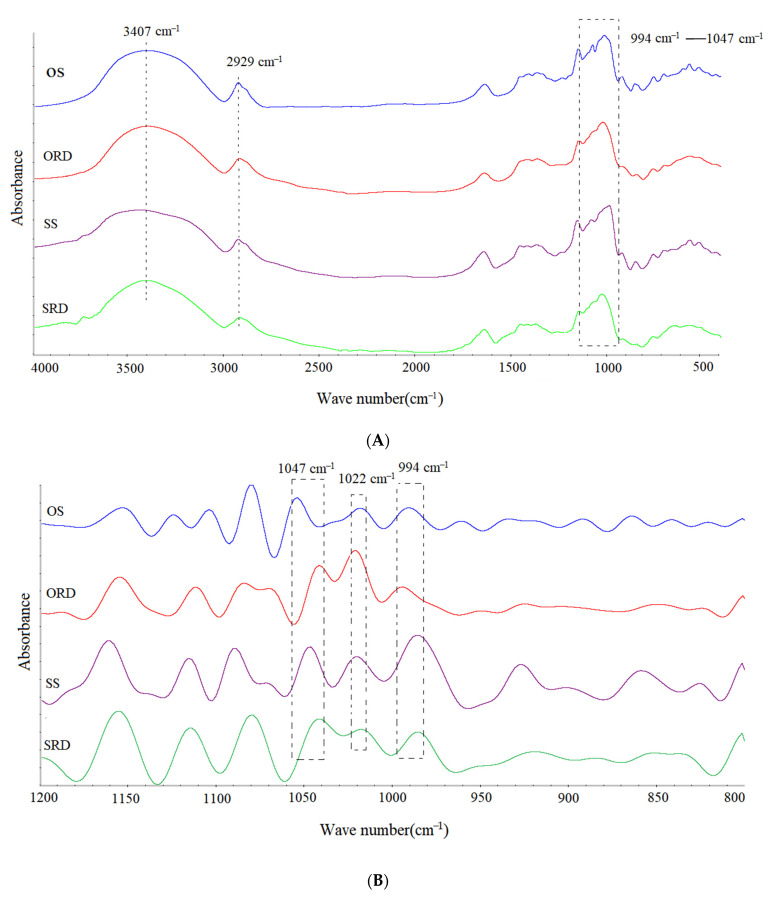
FTIR spectra (**A**) and FTIR spectra after deconvolution (**B**) of sorghum/oat starch and sorghum/oat resistant dextrin. SS: sorghum starch; SRD: sorghum resistant dextrin; OS: oat starch; ORD: oat resistant dextrin.

The FTIR spectra of four samples were recorded to compare the differences in interactions in the structure. The spectra are shown in Figure 2A. The stretching vibration of the hydroxyl group led to a wide absorption peak of approximately 3407 cm^−1^, and the peak width reflected the formation of intermolecular and intramolecular hydrogen bonds. After the preparation process, the -OH peaks of ORD and SRD were lower than those of the original starch, but the peaks of OS and ORD were significantly higher than those of SS and SRD. This may indicate that the prepared RDs retained part of the original water-soluble characteristics of starch and the water solubility of the RDs was related to the water solubility of starch. However, this needs to be confirmed. The peak at 2929 cm^−1^ came from the asymmetric stretching vibration of the C-CH_2_-C of the glucose unit. According to our observations, the bands detected in the spectrum of ORD and SRD were less intense, compared with the native starch, which may be associated with the lower amylose content in the former [21]. The absorbance peaks at 1047, 1022 and 994 cm^−1^ were sensitive to the change in crystallinity, amorphous starch content and water content on intramolecular hydrogen bonds [22]. As shown in Figure 2B, ORD and SRD exhibited a lower strength ratio of 994/1022 cm^−1^ than starches, showing that the preparation reduced the ordered structure of starches. Interestingly, SRD showed an equal strength ratio of 1047/1022 cm^−1^ with SS, while ORD showed a lower strength ratio than OS. This may be due to the more stringent preparation conditions of ORD.

### 3.3. X-ray Ray Diffraction Analysis

**Figure 3:**
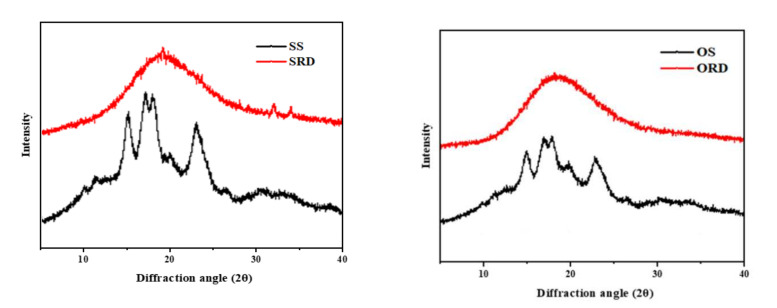
X-ray diffraction patterns of sorghum/oat starch and sorghum/oat resistant dextrin. SS: sorghum starch; SRD: sorghum resistant dextrin; OS: oat starch; ORD: oat resistant dextrin.

As shown in Figure 3, the diffraction characteristic peaks of SS and OS both appear at 15°, 17°, 18°, 20°and 23° (2θ), and the peaks were sharp, indicating that both starches were A-type starches with high crystallinity [23,24]. This result was consistent with the A-type SS obtained by Cabrera-Ramírez et al. [25]. The figure shows that SS had higher crystallinity, which was consistent with the DSC results. However, the XRD patterns of ORD and SRD showed that the sharp peak almost disappeared, and the whole curve became a broad peak. This indicated the serious destruction of the crystalline structure of starch after high-temperature pyrolysis and the crystalline structure of starch was partly lost, increasing the amorphous phase (loss of the peak diffraction).

### 3.4. The WSI and the SP

The WSI was mainly on account of the escape of amylose from swelling particles and the SP reflected the characteristics of amylose. The WSI and the SP reflected the strength of the interaction between starch and water. They were affected by the size, morphology, composition, the molecular weight ratio of amylose/amylopectin and the proportion of long chains in amylopectin. A previous study showed that starch molecules were significantly degraded by heat and acid, as reflected in a continual decrease in the molecular weight of starch, a progressive increase in solubility in water, decreased viscosity in water, and the increased content of free reducing sugar [1]. The impact of temperature on the solubility and the swelling power of four samples is shown in Table 1. The solubility of RDs was very high compared with starch, illustrating that starch molecules were severely degraded in terms of spatial structure and a lot of soluble small molecules were obtained during the dextrinization reaction [6]. This was consistent with the granular morphology. The solubility of ORD was higher than that of SRD at the same temperature. The high solubility indicated that OS degradation was more thorough than SS degradation or that the RD retained part of the original water-soluble characteristics of starch. The swelling power of the two kinds of RD was poor, and the RDs had nearly no effect on the original properties of the food when to food in the form of excipients.

### 3.5. DSC Analysis

Gelatinization temperature was an important index of starch, which was closely related to starch particle crystal structure, particle size and starch composition. DSC was used to obtain the onset temperature (T_O_), the peak temperature (T_P_), the conclusion temperature (T_C_) and the gelatinization enthalpy (ΔH_g_) under different heating conditions, reflecting thermal stability. The T_O_, the T_P_, the T_C_ and the ΔH_g_ of the four samples are presented in Table 2. Compared with OS, SS had a higher melting temperature and a higher enthalpy value: Tc: 75.94 °C vs. 65.43 °C; the ΔH_g_: 14.03 J/g vs. 9.4 J/g. This indicates that SS showed slightly higher thermal stability than OS. Previous studies have found that starches with high amylopectin content and high crystallinity need higher gelatinization temperatures [26,27]. OS was characterized by a high pasting temperature owing to the presence of high amounts of lipids and high relative crystallinity [12]. Similarly, waxy sorghum starch also had a high pasting temperature, due to high amylopectin content. The lower thermal stability in OS compared with SS indicated that the SS has a stronger and more orderly structure. However, there was no phase transition peak in the prepared dextrin. This phenomenon may be related to the loss of the original crystal structure of amylopectin after high-temperature acidolysis, the improvement in the order or crystallinity of the double helix structure in the molecule, or the formation of a new glycosidic bond. This showed that ORD and SRD have good thermal stability when used in food.

### 3.6. In Vitro Digestibility

**Figure 4:**
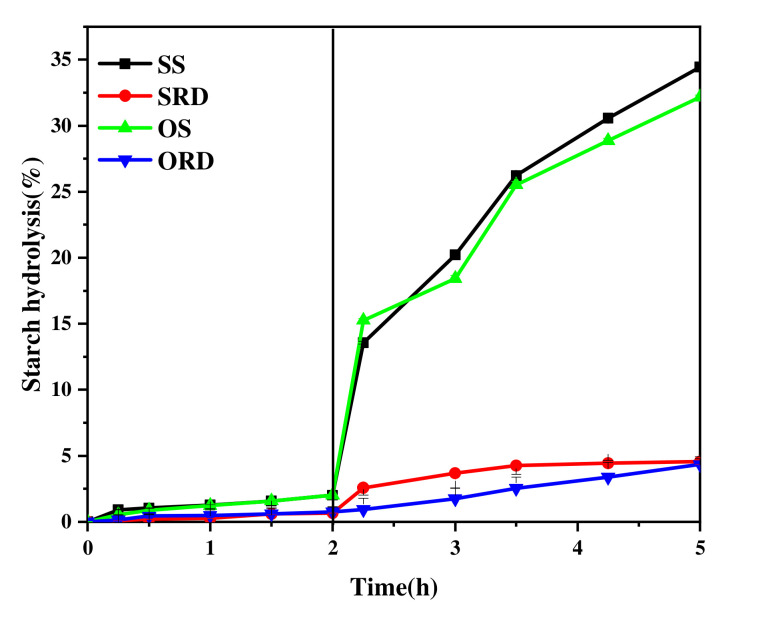
Digestibility changes in sorghum/oat starch and sorghum/oat resistant dextrin. SS: sorghum starch; SRD: sorghum resistant dextrin; OS: oat starch; ORD: oat resistant dextrin.

In vitro starch digestibility changes are shown in Figure 4. In the stage of simulated gastric digestion, the digestibility of the samples was less than 2%. This may be due to the slight hydrolysis of starch by the acidic environment of the stomach. After 3 h of simulated intestinal digestion, the glucose content in the digested products of OS and SS increased significantly, the digestibility of SS increased from 2.02% to 34.23%, and the digestibility of OS increased from 2.02% to 32.16%. The digestibility of ORD and SRD was much lower than that of starch; although it increased slightly, changes were by less than 5%. This was because pancreatin hydrolyzes α-1, 4 and α-1, 6 glycosidic bonds in starch molecules to produce glucose, while the RD formed by starch molecules was a polymer connected by α-1, 2 and α-1, 3 bonds, which was resistant to enzymolysis. In Figure 4, the digestibility of ORD was the lowest. This may have been caused by the smaller particles and more severe pyrolysis conditions of OS, resulting in a greater indigestible fraction. Some studies have shown that the in vitro anti-digestion property of ORD increases with an increase in the intensity of gelatinization conditions [28]. Therefore, ORD and SRD are completely absorbed by the gastrointestinal tract, and the undigested carbohydrates enter the large intestine and are fermented by the Lactobacillus in the large intestine.

### 3.7. Probiotics Effect In Vitro

The effects of RD digesta on the proliferation of two different strains are shown in Table 3. According to our results, both ORD and SRD promote the proliferation of three different strains. It was suggested that they have a prebiotic effect and promote the growth and reproduction of intestinal probiotics. We found that different amounts of resistant dextrin had different probiotic effects on the three bacteria. Increasing RD concentration, first increases proliferation, which then decreases over a concentration range of 0 mg/mL to 20 mg/mL, while the total colony number still increases compared with the control. The most suitable amount of dextrin to add differs for different probiotics. Compared with the digesta of SRD, the digesta of ORD contributed more effectively to the proliferation of both Lactiplantibacillus plantarum and Lactobacillus acidophilus. However, SRD has a good proliferative effect on Lactobacillus delbrueckii, which highlights possible correlations between structural features and prebiotic activity. However, this needs to be confirmed. For Lactiplantibacillus plantarum, the best effect was found in ORD at a concentration of 16 mg/mL (*p* < 0.05), with the counts increasing by 8.08 log CFU/mL compared with SRD, and the counts increased by 8.89 log CFU/mL compared with the control; for Lactobacillus acidophilus, the counts increased by 7.80 log CFU/mL compared with SRD at an ORD concentration of 12 mg/mL, and the counts increased by 8.74 log CFU/mL compared with the control. For Lactobacillus delbruecki, SRD was most effective at a concentration of 16 mg/mL (*p* < 0.05), with the counts increasing by 8.72 log CFU/mL compared with the control. Similar results were obtained with potato resistant dextrin by supporting the growth of probiotics (Bifidobacterium and Lactobacillus) in a comparative study reported by Barczynska et al. [29].

**Figure 5:**
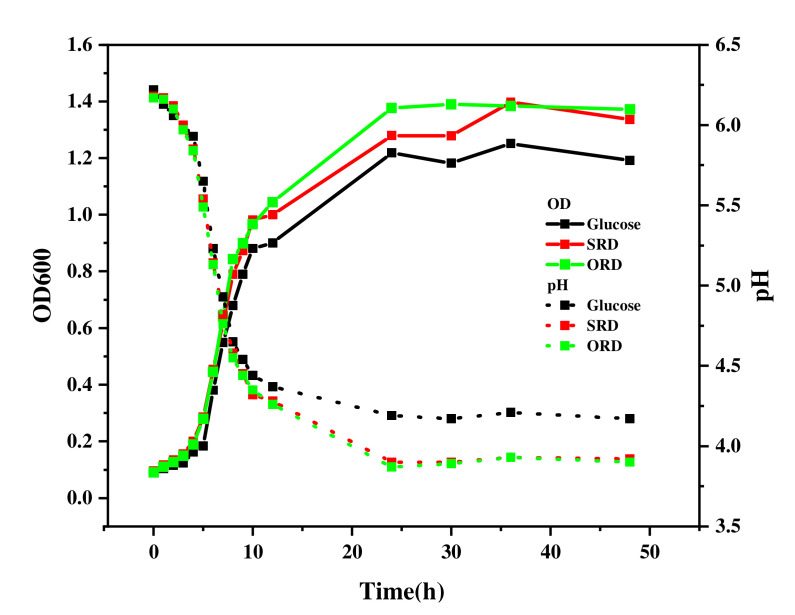
OD_600_ and change in pH of *Lactiplantibacillus plantarum* cultured with different substrates. SRD: sorghum resistant dextrin was used as a substrate; ORD: oat resistant dextrin was used as a substrate; glucose: control group.

OD_600_ and change in pH of *Lactiplantibacillus plantarum* cultured with different substrates are shown in Figure 5. By culturing *Lactiplantibacillus plantarum* with ORD, SRD and glucose, and monitoring the OD_600_ and pH, we found that the OD_600_ and the pH of the three cultures tended to be stable after 24 h of culture. With the change in time, the OD_600_ of each test group first increased sharply, then increased steadily, and finally tended to be stable. The pH also became lower and lower with the passage of time, and finally stabilized. Compared with the glucose group, the OD_600_ of the test group supplemented with RDs were higher than those of the glucose group at 48 h. The pH of the glucose group, SRD and ORD were 4.17, 3.92 and 3.90, respectively. This showed that ORD had the strongest prebiotic effect and produced the most acid. This phenomenon also proved once again that SRD and ORD had a better prebiotic effect.

## 4. Conclusions

In conclusion, ORD and SRD possessed irregular apparent morphology, low crystallinity and a disordered internal structure, but showed good hydration characteristics and thermal stability, compared with their original starch. In addition, both ORS and SRD acquired indigestibility and obvious prebiotic effects demonstrated by the promoting effects on the growth of three *Lactobacillus—Lactiplantibacillus plantarum*, *Lactobacillus* *acidophilus* and *Lactobacillus* *delbrueckii*. ORD had a better promoting effect on *Lactiplantibacillus plantarum* and *Lactobacillus acidophilus*, while SRD had a better promoting effect on *Lactobacillus delbrueckii*. These results enrich the research and development of products rich in prebiotics, and promote the high-value utilization of sorghum and oat.

## Figures and Tables

**Table 1:** The solubility and expansibility of sorghum/oat starch and sorghum/oat resistant dextrin.

Temperature (°C)	WSI (g·100 g^−1^)				SP (g·100 g^−1^)			
	SS	SRD	OS	ORD	SS	SRD	OS	ORD
4	3.71 ± 0.05 ^a^	90.32 ± 0.12 ^a^	4.19 ± 0.02 ^a^	91.50 ± 0.21 ^a^	1.03 ± 0.05 ^a^	2.15 ± 0.09 ^a^	1.00 ± 0.02 ^a^	1.12 ± 0.10 ^a^
25	3.72 ± 0.02 ^a^	91.21 ± 0.22 ^b^	4.25 ± 0.09 ^a^	91.66 ± 0.25 ^a^	1.03 ± 0.02 ^a^	2.45 ± 0.11 ^b^	1.00 ± 0.09 ^a^	1.26 ± 0.08 ^a^
37	3.90 ± 0.04 ^b^	92.15 ± 0.13 ^c^	5.56 ± 0.01 ^b^	92.21 ± 0.16 ^b^	1.02 ± 0.04 ^a^	2.64 ± 0.08 ^b^	1.02 ± 0.01 ^a^	1.37 ± 0.14 ^ab^
100	17.84 ± 0.10 ^c^	92.65 ± 0.21 ^d^	20.72 ± 0.06 ^c^	92.87 ± 0.14 ^c^	16.48 ± 0.10 ^b^	3.21 ± 0.12 ^c^	15.45 ± 0.06 ^b^	1.58 ± 0.12 ^b^

The data are the means of three independent experiments ± standard deviations (*n* = 3). Values with different superscript letters in the same column (lowercase letters) differ significantly (*p* < 0.05). SS: sorghum starch; SRD: sorghum resistant dextrin; OS: oat starch; ORD: oat resistant dextrin.

**Table 2:** DSC profiles of sorghum/oat starch and sorghum/oat resistant dextrin.

Samples	T_o_/°C	T_P_/°C	T_C_/°C	ΔH_g_/J·g^−1^
SS	65.01 ± 0.11 ^a^	69.89 ± 0.07 ^a^	75.94 ± 0.56 ^a^	14.03 ± 0.54 ^a^
SRD	——	——	——	——
OS	57.30 ± 0.01 ^b^	61.10 ± 0.05 ^b^	65.43 ± 0.03 ^b^	9.40 ± 0.31 ^b^
ORD	——	——	——	——

The data are the means of three independent experiments ± standard deviations (*n* = 3). Values with different superscript letters in the same column (lowercase letters) differ significantly (*p* < 0.05). “——” not available. SS: sorghum starch; SRD: sorghum resistant dextrin; OS: oat starch; ORD: oat resistant dextrin.

**Table 3:** Effects of resistant dextrin digesta on the proliferation of Lactobacillus plantarum, Lactobacillus acidophilus and Lactobacillus delbrueckii ((X ± S) log CFU/mL).

Concentration (mg/mL)		*Lactobacillus plantarum*	*Lactobacillus acidophilus*	*Lactobacillus delbrueckii*
		log (CFU/mL)		
SRD	0	8.87 ± 0.01 ^a^	8.91 ± 0.01 ^a^	8.83 ± 0.01 ^a^
	4	9.09 ± 0.02 ^d^	8.93 ± 0.01 ^ab^	8.90 ± 0.01 ^cd^
	8	9.15 ± 0.01 ^g^	8.97 ± 0.01 ^c^	8.98 ± 0.01 ^fg^
	12	9.09 ± 0.02 ^d^	9.01 ± 0.02 ^ef^	9.03 ± 0.01 ^h^
	16	9.05 ± 0.01 ^c^	9.11 ± 0.01 ^i^	9.08 ± 0.01 ^i^
	20	9.04 ± 0.02 ^bc^	9.06 ± 0.01 ^g^	8.97 ± 0.01 ^ef^
ORD	0	8.87 ± 0.01 ^a^	8.91 ± 0.01 ^a^	8.83 ± 0.01 ^a^
	4	9.10 ± 0.01 ^de^	8.99 ± 0.01 ^de^	8.88 ± 0.01 ^bc^
	8	9.13 ± 0.01 ^fg^	9.09 ± 0.01 ^h^	8.96 ± 0.01 ^e^
	12	9.14 ± 0.01 ^g^	9.13 ± 0.01 ^j^	9.00 ± 0.02 ^g^
	16	9.18 ± 0.01 ^h^	9.06 ± 0.01 ^g^	9.04 ± 0.01 ^h^
	20	9.11 ± 0.01 ^ef^	8.98 ± 0.01 ^cd^	8.99 ± 0.01 ^fg^

The data are the means of three independent experiments ± standard deviations (*n* = 3). Values with different superscript letters in the same column (lowercase letters) differ significantly (*p* < 0.05). SRD: sorghum resistant dextrin was used as a substrate; ORD: oat resistant dextrin was used as a substrate.

## Data Availability

All data are provided in the manuscript.
